# Integrating Domain Knowledge Into Deep Networks for Lung Ultrasound With Applications to COVID-19

**DOI:** 10.1109/TMI.2021.3117246

**Published:** 2021-10-04

**Authors:** Oz Frank, Nir Schipper, Mordehay Vaturi, Gino Soldati, Andrea Smargiassi, Riccardo Inchingolo, Elena Torri, Tiziano Perrone, Federico Mento, Libertario Demi, Meirav Galun, Yonina C. Eldar, Shai Bagon

**Affiliations:** Weizmann Institute of Science 34976 Rehovot 7610001 Israel; Weizmann Artificial Intelligence Center (WAIC)Weizmann Institute of Science 34976 Rehovot 7610001 Israel; Department of Computer ScienceThe Hebrew University of Jerusalem 26742 Jerusalem 9190501 Israel; Beilinson HospitalRabin Medical Center 36632 Petah 49100 Israel; Sackler Faculty of MedicineTel Aviv University 26745 Tel Aviv 6997801 Israel; Valle del Serchio General Hospital 55100 Lucca Italy; Fondazione Policlinico Universitario A. Gemelli IRCCS 00168 Rome Italy; Humanitas Gavazzeni 18505 24125 Bergamo Italy; Fondazione IRCCS Policlinico San Matteo di Pavia 27100 Pavia Italy; Department of Information Engineering and Computer ScienceUniversity of Trento 19034 38123 Trento Italy

**Keywords:** COVID-19, deep learning, image classification, lung ultrasound, semantic segmentation

## Abstract

Lung ultrasound (LUS) is a cheap, safe and non-invasive imaging modality that can be performed at patient bed-side. However, to date LUS is not widely adopted due to lack of trained personnel required for interpreting the acquired LUS frames. In this work we propose a framework for training deep artificial neural networks for interpreting LUS, which may promote broader use of LUS. When using LUS to evaluate a patient’s condition, both anatomical phenomena (e.g., the pleural line, presence of consolidations), as well as sonographic artifacts (such as A- and B-lines) are of importance. In our framework, we integrate domain knowledge into deep neural networks by inputting anatomical features and LUS artifacts in the form of additional channels containing pleural and vertical artifacts masks along with the raw LUS frames. By explicitly supplying this domain knowledge, standard off-the-shelf neural networks can be rapidly and efficiently finetuned to accomplish various tasks on LUS data, such as frame classification or semantic segmentation. Our framework allows for a unified treatment of LUS frames captured by either convex or linear probes. We evaluated our proposed framework on the task of COVID-19 severity assessment using the ICLUS dataset. In particular, we finetuned simple image classification models to predict per-frame COVID-19 severity score. We also trained a semantic segmentation model to predict per-pixel COVID-19 severity annotations. Using the combined raw LUS frames and the detected lines for both tasks, our off-the-shelf models performed better than complicated models specifically designed for these tasks, exemplifying the efficacy of our framework.

## I. Introduction

The diagnosis and treatment of respiratory diseases rely on the use of various imaging modalities. Chest CT is considered the imaging gold standard for pulmonary diseases [1], [2]; however, it is expensive and non-portable. Another standard imaging modality utilized to investigate the lung is chest X-ray [3]. Both modalities involve ionizing radiations, which are potentially harmful to the patient. This is particularly significant for specific patient populations such as children, pregnant women, and patients who require repeated examinations over a short period of time. Moreover, CT is generally not available in every hospital nor applicable at bedside, thus requiring patients’ mobility. When dealing with a highly infectious disease, this last aspect further increases the risk of contamination within the hospital. Compared to these imaging technologies, ultrasound imaging is safer, cost-effective, more widely available, and transportable, thus has the potential of reaching a much larger population, including non-hospitalized patients. More importantly, there is growing evidence showing that lung ultrasound (LUS) can be effectively used as an imaging modality for pulmonary diseases (e.g., [4]–[6]).

The recent outbreak of COVID-19 pandemic drove clinicians to use LUS imaging also in emergency rooms [7]. Findings suggest LUS can assist in both detecting COVID-19 patients and monitoring their condition throughout their hospitalization [6], [8]–[11]. However, LUS provides only local information on the state of the lung surface. Therefore, it is crucial to carefully define the required amount and distribution of scanning areas. To this end, multi-centre studies aimed at finding the optimal trade-off between a fast and accurate examination were performed. In the context of COVID-19, a 12-area approach was suggested to represent an optimal trade-off between accuracy, timing and examination complexity [12], [13].

**Fig. 1.**
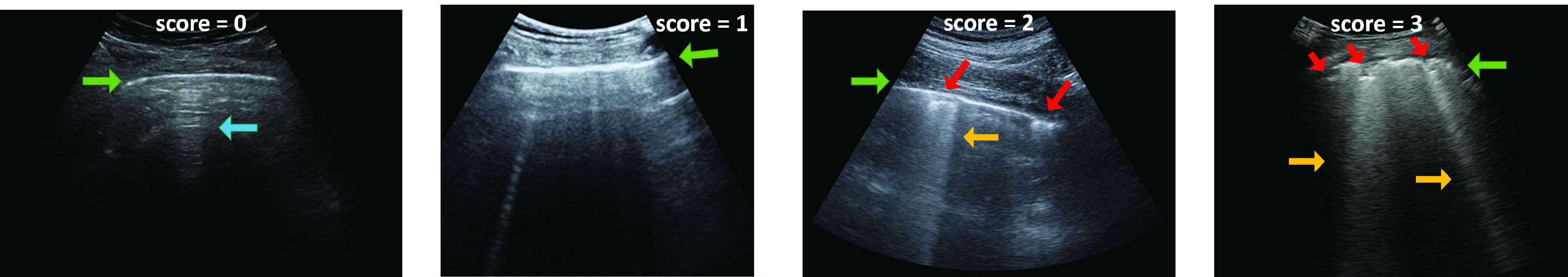
**COVID-19** **Severity scores.** LUS frames exemplifying the severity score of [6] from healthy (score $=0$, left) to severe (score $=3$, right). One can observe the peural line (green), A-lines (blue), subpleural consolidations (red) and vertical artifacts (e.g., B-lines and “white lung”) (yellow). While the pleural line and consolidations are anatomical features, the A-lines and vertical artifacts are sonographic echoes.

When using LUS to evaluate a patient’s condition, both anatomical findings (e.g., presence of consolidations, the integrity of the pleural line [14]), as well as sonographic artifacts (such as A-lines and B-lines [15]) are of importance. Examples of these phenomena are shown in Fig. 1. However, spotting these findings and correctly interpreting them requires highly trained personnel. Consequently, to date, LUS is not widely adopted as its potential would reasonably suggest, particularly in the face of dire needs arising in treating patients with the COVID-19 pandemic.

Deep neural networks (DNN) and deep learning (DL) proved to be very powerful tools for accomplishing many challenging tasks, especially in the domain of image understanding. Given enough training examples ( $>{millions}$) and computational resources, deep models can even exceed human performance on specific tasks (e.g. [16]–[18]). There is also growing work on applying DNNs to ultrasound imaging (see [19] and references therein). Nonetheless, when training data is hard to come by, as is often the case with medical imaging, it becomes more challenging to successfully train these complex models. One approach to combat limited training data is to use domain knowledge to constraint the space of learned models. These approaches lead to various forms of model-based learning [20], [21], resulting with task-specific architectures.

In this work we take a different path for overcoming the challenge of limited training data. Instead of designing task-specific DNNs for LUS analysis, we propose a framework that integrates LUS domain knowledge into the *inputs* used by standard DNNs. We explicitly enrich the input to the model with domain specific knowledge. Specifically, we suggest to inform the model of important anatomical features and sonographic artifacts. We detect the pleural line and vertical artifacts (such as B-lines, “white lung” etc.) as a preprocessing stage. This automatically extracted domain-specific information is then fed, as additional input channels, much like RGB color channels in “natural images”, to a DL model alongside the raw LUS frame. These domain-specific channels allow the model to better tune and attend to relevant features and findings characteristic of this specific domain. This approach for utilizing domain knowledge puts the focus on data preparation and alleviates the need to design task-specific DNNs. A similar approach, i.e., augmenting the raw input with additional masks, for analysing chest Xray of COVID-19 patients was proposed in [22].

**Fig. 2.**
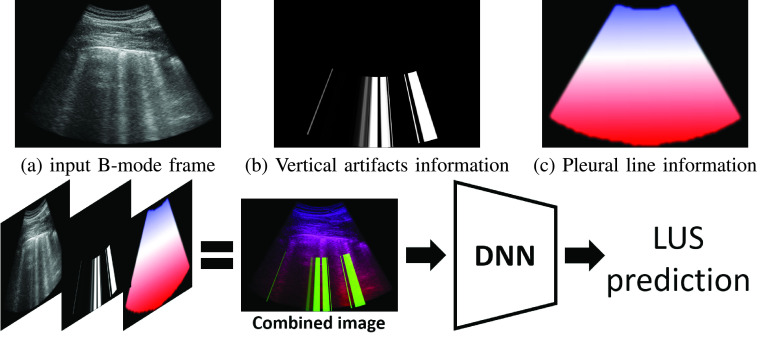
**Our framework for integrating domain knowledge into deep neural networks (DNN) for LUS.** Top: Input frame (a) is augmented with two additional channels containing LUS domain specific knowledge: (b) Automatically detected vertical artifacts (e.g., B-lines, “white lung”). (c) A signed distance mask from the pleural line. Bottom: The concatenation of these three channels (viewed as RGB image) are used as input for the DNN, enhancing the relevant frame regions.

Fig. 2 illustrates our approach: Fig. 2 (top) shows an example of an input LUS frame and the automatically detected vertical artifacts and pleural line channels. The resulting concatenation of these masks and the raw input frame is then used as an input to a standard DNN model (bottom of Fig 2). Explicitly providing the model with this automatically extracted domain knowledge allows using simple off-the-shelf image classification neural network architectures, and rapidly and efficiently finetuning them to perform well on LUS data. Our framework allows to effectively and efficiently train DNN on LUS data, even when only several thousands of training examples are available. Moreover, we train a *single* task-specific DNN model capable of handling LUS frames acquired by either convex or linear probes.

We demonstrate the efficacy of our framework on COVID-19 severity assessment, both on LUS frame classification as well as the task of semantic segmentation. We evaluated our proposed framework using the ICLUS dataset curated by the Ultrasound Laboratory Trento, Italy [23]. We finetuned simple image classification models on the combined raw LUS frames and the detected lines. We also finetune a semantic segmentation model to predict per-pixel COVID-19 annotations. Our finetuned off-the-shelf models performs better than complicated models specifically designed for these tasks.

To summarize, in this work we make the following contributions:

(a) A widely applicable framework for incorporating LUS domain-specific knowledge into deep neural networks.
(b) A unified framework capable of handling LUS frames acquired by either linear or convex probes.
(c) Exceeding state of the art results on the ICLUS COVID-19 severity prediction benchmark, both for LUS frame classification and semantic segmentation.

This paper is organized as follows: the guiding principles of our framework are outlined in §II, while the specific details of our implementation are provided in §III. We exemplify the efficacy of our framework training DNNs to perform LUS frame classification in §IV and on the task of semantic segmentation in §V. We conclude in §VI.

## II. Method

**Fig. 3.**
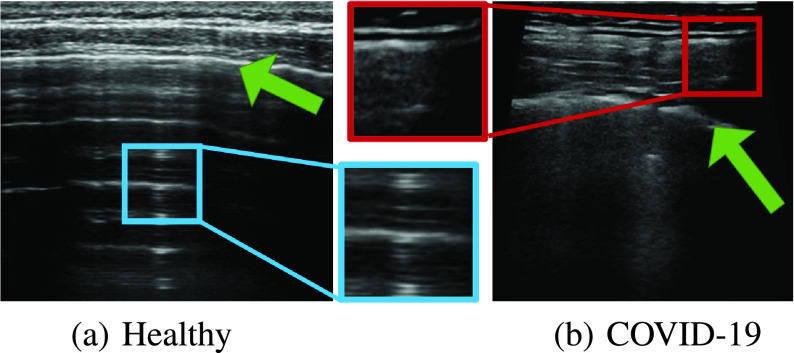
**Relative location and interpretation.** Visually similar regions in LUS frames may account for very different findings if located above the pleural line (green arrow) or below it. For instance, the blue region *below* the pleural line shows A-lines, while the red region *above* the pleural line shows muscle tissue.

LUS frames have a strong artefactual nature. The pleural line partitions the frame into two parts: the top part showing the exterior tissue and the bottom part showing the aerated lung cavity. Due to the dramatic difference in their acoustical properties, these two regions appear quite differently in LUS. Moreover, this change in acoustical conditions gives rise to sonographic artefacts such as A-lines, B-lines and “white lung”. When interpreting LUS one needs to take these unique characteristics into account: For instance, bright horizontal lines can be A-lines if they are *under* the pleural line (Fig. 3 blue), but may account for a completely different findings if they are observed *above* the pleural line (Fig, 3 red), leading to a radically different interpretation. While the presence of A-lines usually suggests healthy condition (Fig. 3a), observing these bright lines above the pleural line may lead to erroneous assessment for the COVID-19 patient in Fig. 3b.

DNN architectures designed for image analysis tasks are oblivious to these idiosyncrasies of LUS. Our framework proposes to make DNNs aware of these idiosyncrasies not by changing their design, but rather by highlighting relevant and salient features via preprocessing of the input LUS frames. Specifically, we propose to augment the raw input LUS frame with additional channels, each channel highlights domain specific information. Fig. 2 illustrates our approach: the input raw frame (a), is augmented with additional channels highlighting vertical artifacts (b) and the location of the pleural line (c).

Our framework for incorporating domain knowledge as additional special input channels is not restricted to any number of additional channels or any specific choice of channels. In this work we made a design choice, selecting only two additional input channels: one highlighting the vertical artifacts and the other the location of the pleural line.

As noted, the pleural line plays a key role in interpreting LUS. Therefore, we choose to encode the pleural line information by measuring the *signed* distance of each pixel to the pleural line: negative distance above the pleural line (Fig 2c, blue shade), and positive below (Fig 2c, red shade). Integrating the signed distance into the input channels allows the network to trivially distinguish between the exterior tissue (upper part of the frame, negative signed distance), the lung cavity (lower part of the frame, positive signed distance), and the pleural line region (small distance). Another important phenomenon unique to LUS are vertical artifacts, such as B-lines and “white lung”, indicating loss of aeration of the lung. Their presence usually indicate a pathological condition. We therefore add another channel with a segmentation mask indicating possible locations of vertical artifacts (Fig. 2b). We concatenate the two masks as the second and third channels on top of the original gray-level channel of the input LUS frame. The resulting 3-channel image efficiently encodes LUS-specific information allowing for training DNN models to perform LUS-specific predictions as shown in the bottom of Fig. 2. This multi-channel representation is applicable for LUS frames obtained by either convex or linear probes, allowing to train a *single* task specific DNN capable of handling convex as well as linear frames.

To summarize, our framework preprocesses a LUS frame into a three channel image, similar in structure to an RGB natural image. These additional input channels represent LUS domain specific expert knowledge. Training DNNs for various LUS tasks is more efficient using these additional channels. Our framework thus consists of the following steps:

*Preprocessing stage:* extract vertical artifacts and pleural line masks from LUS frames, and combine them as additional input channels.
*DNN training stage:* train a DNN on the combined 3-channel input frames.

Our approach to processing LUS is applicable to many LUS frame analysis applications and tasks. It does not dictate the use of any specific DNN architecture. Moreover, it handles LUS frames obtained by either convex or linear probes in a unified manner. Indeed, we demonstrate our framework on two different tasks: classification and semantic segmentation of COVID-19 severity assessment, where for each task we train *one* DNN model for both convex and linear frames.

## III. Implementation Details

In the previous section we outlined the concepts on which our framework is based. This section describes a practical implementation of our framework.

### A. Vertical Artifacts Estimation

Robustly and accurately detecting vertical artifacts (i.e., B-lines) is a challenging task [15], [24], [25]. Existing approaches were either probe-type specific [24], [25] or requiring LUS videos [26]. Here, we do not aim to perfectly solve it, but only to steer the downstream DNN model in the right direction. Therefore, we resort to a simplistic approach, that is fast, unsupervised and provides an informative, albeit noisy, estimation of vertical artifacts.

According to recent developments in LUS [27], [28], vertical artifacts are sonographic signs caused by complex interaction of the multiple scattering phenomena that may form in the presence of an alteration occurring at the lung surface. When forming the LUS frame, the ultrasound signals produced by the multiple scattering events, in case of resonance phenomena, are then interpreted as a bright-vertical-line emitting from the pleural line and aligned along the ultrasound beam axis.

**Fig. 4.**
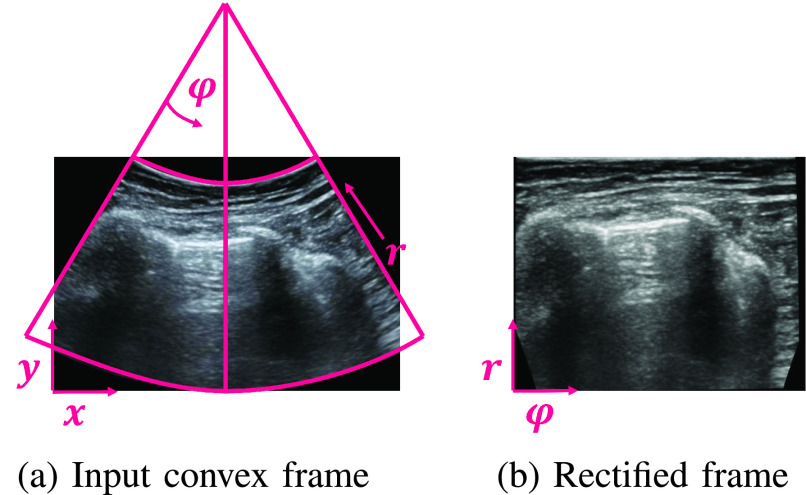
**Rectifying convex frames.** (a) Original frame in Cartesian ${x}$- ${y}$ coordinates and the induced polar ${r}$- $\varphi $ coordinates. (b) The rectified frame according to its polar coordinate system. The transformation from one coordinate system to the other is invertible given the focal point of the transducer. This process is detailed in the Appendix.

**Fig. 5.**
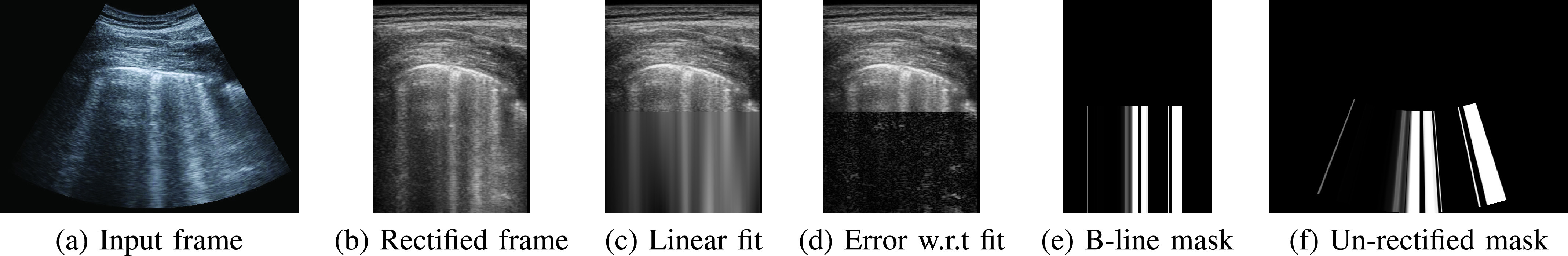
**Detecting vertical artifacts as bright columns.** (a) Input. (b) Rectified convex frame according to its polar coordinates (Fig. 4). (c) Fit of the intensities of the lower half of each column with a linear function: ${a}_{x}\cdot {y} + {b}_{x}$. (d) Error between the actual intensity and the linear fit. B-lines, “white lung” and similar vertical artifacts have low error. (e) Columns whose linear fit is above threshold $\tau _{\textit {bright}}$ and the error is below threshold $\tau _{\textit {err}}$ are marked as vertical artifacts. (f) Un-rectify masks of convex frames back to their Cartesian coordinates.

Our approach is based on the observation that the orientation of the vertical artifacts is known and depends only on the probe type used. For a linear probe, these artifacts are exactly vertical. For convex probes, the orientation of these artifacts depends on their polar coordinate: the further away they are from the center of the frame, the more tilted towards the outside they are. Consequently, if we rectify a convex frame according to the polar coordinates induced by the convex probe (see Fig. 4) all vertical artifacts will become strictly vertical, as in frames captured using linear probes. This phenomenon may be observed in Fig. 5a, where the orientation of the vertical artifacts vary according to their polar coordinate. Fig. 5b shows the rectified frame. Note how all these artifacts are now vertical. The rectification process is detailed in the Appendix.

The canonical orientation of the vertical artifacts allows us to detect them quite easily. Let $I_{xy}$ be the intensity at the $(x, y)$ coordinate. We look for columns at the lower half of the frame that are bright and have relatively low noise. To find such columns we perform the following steps:

**Linear fit:** For each column, $x$, we fit its intensities, $I_{xy}$, with a linear function of the vertical coordinate. That is, we estimate scalars slope, $a_{x}$, and intercept $b_{x}$ for each column $x$ that minimizes the squared error between a linear function of the $y$ coordinate, $a_{x}\cdot y + b_{x}$, and the actual intensity of the pixel $I_{xy}$: $\arg \min _{a_{x},b_{x}}\sum _{y}\left \vert{ a_{x}\cdot y + b_{x} - I_{xy}}\right \vert ^{2}$. Fig. 5c shows the linear fit of the intensities for each column.

**Error estimation:** We then compute the error between the fit and the actual pixel value, $\sum _{y}\left \vert{ a_{x}\cdot y + b_{x} - I_{xy} }\right \vert $ (Fig. 5d). Vertical artifacts have relatively low error, as opposed to consolidations, speckles or columns intersecting with A-lines.

**Thresholding:** All columns whose linear fit is above threshold $\tau _{bright}$ and the error is below threshold $\tau _{err}$ are marked as vertical artifacts (Fig 5e). We empirically determined the values of the thresholds based on a small set of representative frames sampled from the training set.

The resulting binary mask is then transformed back from polar coordinates to the original Cartesian coordinates to form the vertical artifacts mask for the input frame (Fig. 5f). Note that for frames captured using linear probes we do not need to change to polar coordinates, and can do the same processing in the original Cartesian coordinate system.

It is important to note that although the vertical artifacts appear in a more consistent manner in the polar coordinates for convex frames, the actual underlying anatomy (e.g., ribs, pleura etc.) is *distorted*: the beamforming process is aimed at representing the true underlying anatomy in the *original Cartesian* coordinates. Therefore, we only use the polar coordinates to estimate the vertical artifacts, but maintain all other analysis and processing in the original Cartesian coordinates, as they authentically reflect the underlying anatomy.

### B. Pleural Line Detection

The pleural line is a bright thin line that roughly crosses the frame from side to side (See Fig. 3 and Fig. 1 green). A straightforward approach for pleural line detection was proposed in [24]. They use the Radon transform of the frame and apply an iterative optimization process to locate a distinct and significant bright horizontal line in the Radon space.

Once we obtain the location and orientation of the pleural line from [24] we applied a signed distance transform. We further scale the signed distance such that the distance from the pleural line to the bottom of the frame is equal 1. An example of a pleural line mask is shown in Fig 2c: negative distance at the top part of the frame and positive distance at the bottom.

Both approaches for vertical artifacts and pleural line detection are simple but inexact. Nevertheless, the domain knowledge they extract is introduced in a “soft” manner to the network via additional input channels. This way the network is trained to reason with this noisy data and distil meaningful cues from it to facilitate better performance on any down-stream target LUS task.

### C. DNN Models

Our framework is not restricted to any specific deep neural network architecture. In fact, working with three input channels (i.e., the raw frame, vertical artifacts mask and pleural signed-distance) allows us to use models pre-trained on natural 3-channel RGB images “as-is”. This gives us the flexibility to opt for large models (e.g., ResNet-18 [29]) when accuracy is of importance, or trade it for a light-weighted model (e.g., MobileNetV2 [30]) when computing resources are scarce. For the semantic segmentation task we used the DeepLabV3++ [31] model.

All these models were pre-trained on natural RGB images to perform image classification [32] or semantic segmentation [33], and their trained weights are readily available on-line. Once we choose our model, we can use its pre-trained weights except of the last task-specific prediction layer that needs to be trained from scratch.

### D. Finetuning the Models

To make our trained model more robust to small changes in the input frames we applied various augmentations to the training data. The set of augmentation functions, each applied with a randomly sampled strength bounded by a set maximum, consists of: affine transformations (translation (max. $\pm 10\%$), rotation (max. $\pm 23^\circ $), scaling (max. 10%)), horizontal flipping (50%). We additionally applied random jittering to the raw gray-level frame channel (contrast (max $\pm 30\%$), brightness (max $\pm 30\%$)). These augmentations were verified by [23] to be clinically meaningful, e.g., a LUS frame rotated by up to 23° is still considered a plausible LUS frame. To encourage the model to be more robust to the exact location of the pleural line we applied a random global shift to the signed distance channel (max $\pm 8\%$). We used the same augmentations for the image classification and the semantic segmentation tasks.

We implemented our framework using pytorch [34] and used the supplied torchvision.models and their pretrained weights. We finetuned the models using Adam optimizer [35]. We used a fixed learning rate of $\lambda = 0.0075$ ( $1e-4$ for the semantic segmentation task) and default values $\beta _{1}=0.9$ and $\beta _{2}=0.999$.

We used the same loss functions as [23]: the Soft ORDinal (SORD) loss for the classification task and cross-entropy loss for the semantic segmentation task.

## IV. COVID-19 Severity Grading Results

We evaluated our proposed framework on the task of COVID-19 severity grading. It has been recently shown that LUS can be used for stratification and monitoring of patients with COVID-19 [6], [10]. Soldati *et al.* [6] proposed a 4-level scoring system with scores ranging from 0 to 3. Score 0 indicates a healthy lung characterised by a continuous pleural-line and visible A-lines artifacts. In contrast, score 1 indicates first signs of abnormality mostly related to small alterations in the pleural-line, and the appearance of few vertical artifacts. Scores 2 and 3 are representative of a more advanced pathological state, with the presence of small or large consolidations, respectively, and significant presence of vertical artifacts (B-lines and “white lung”). Fig. 1 shows example frames representative of each score. This scoring system is the only imaging protocol and scoring system specific to COVID-19. It has been validated against other imaging protocols [13] and there is evidence of its prognostic value [11].

We use our framework to train a deep model to classify LUS frames to their annotated COVID-19 severity score, and compare the performance of models trained in our framework to previously published results.

### A. Data

We use the ICLUS dataset [23] curated by the Ultrasound laboratory in Trento, Italy.^1^ The ICLUS dataset contains 277 LUS videos of 35 patients, from 5 different medical centers with total of 58,924 frames, out of which 45,560 frames acquired using convex probes and 13,364 frames acquired using linear probes.^1^https://iclus-web.bluetensor.ai/

All frames in the ICLUS dataset were carefully and manually annotated into one of the four severity scores. The annotations were additionally verified by expert clinicians. Nevertheless, Roy *et al.* [23] reported only 67% agreement across annotators per LUS video, emphasising the difficulty of the task. The ICLUS dataset is then split into a train and test set, with the test set comprising of 10,709 frames. The split is performed at patient level: data from any patient is either in the train or test set, but not in both. More details about the ICLUS dataset can be found in [23].

As a pre-processing stage, we computed vertical artifacts masks and pleural line signed distance masks, as described in §III-A and §III-B. We concatenate these two masks to the original input frame to form an input tensor with 3 channels.

### B. Results

We used our framework to finetune a ResNet-18 model to classify each frame to its annotated severity score. We measured the performance of models trained using our framework in terms of F1-score, which is the harmonic mean of precision and accuracy. Similar to [23] we used two settings: *Setting 1* considers the F1 score computed on the entire test set. *Setting 2* considers the F1 score computed on a modified version of the test set obtained by dropping, for each video, the $K$ frames before and after each transition between two different ground truth scores, potentially removing ambiguous frames, thereby allowing us to identify the impact of noisy labeling on the performance of the model. Table I compares our results ( $3^{rd}$ row) to the results of Roy *et al.* [23] (rows 1 and 2) using the two settings.

**TABLE I:** Quantitative Results: F1 Scores for Per-Frame COVID-19 Severity Classification

	Model			Input channels			Settings1	Settings2 Drop Transition Frames (K)			
							All Frames	$\text{K} = 1$	$\text{K}=3$	$\text{K}=5$	$\text{K}=7$
1	ResNet-18 (Roy etal.)			✓			62.2	63.9	65.5	66.9	67.8
2	CNN-Reg-STN (Royetal.)			✓			65.1	66.7	68.3	69.5	70.3
3	Ours	Ablation	Resnet-18	✓	✓	✓	68.8	70.2	72.4	73.9	75.2
4			ResNet-18	✓		✓	64.5	65.7	67.6	69.0	70.1
5			ResNet-18	✓	✓		64.7	66.1	68.5	70.3	71.8
6			ResNet-18		✓	✓	45.1	45.7	46.7	47.4	47.6
7		Ablation	VGG 16	✓	✓	✓	65.7	67.0	69.0	70.6	72.0
8			VGG 16	✓		✓	63.4	64.6	66.2	67.3	68.1
9			VGG 16	✓	✓		62.5	64.0	66.3	68.2	69.6
10			VGG 16	✓			61.8	62.8	64.4	65.5	66.5
11		Ablation	MobileNet V2	✓	✓	✓	67.3	68.9	71.2	72.9	74.6
12			MobileNet V2	✓		✓	65.8	67.0	68.5	70.0	70.5
13			MobileNet V2	✓	✓		64.6	65.9	67.9	69.4	70.5
14			MobileNet V2	✓			60.0	60.6	62.0	63.2	64.5
15		Ablation	MobileNet V3	✓	✓	✓	66.3	67.6	69.6	70.9	72.0
16			MobileNet V3	✓		✓	64.6	65.8	67.7	69.1	70.2
17			MobileNet V3	✓	✓		64.6	65.9	67.9	69.5	71.1
18			MobileNet V3	✓			61.5	62.6	64.4	65.7	67.0
19		Ablation	ResNet-101	✓	✓	✓	62.2	63.3	64.9	65.9	66.4
20			ResNet-101	✓		✓	60.1	61.5	63.4	65.0	66.4
21			ResNet-101	✓	✓		61.3	62.5	64.3	65.7	67.1
22			ResNet-101	✓			59.4	60.5	61.9	62.8	63.6

Using the same ResNet-18 architecture our model achieved $\text{F}1=68.8$% score compared to $\text{F}1=62.2$% achieved by the same architecture, but without the explicit use of the vertical artifacts and pleural line information. Moreover, our ResNet-18 model outperforms the CNN-Reg-STN architecture proposed by [23] that was specifically designed to cope with the idiosyncrasies of LUS data. We see that it is more advantageous to incorporate domain knowledge as additional input channels than as deep neural architecture designs. We also presented results of $\text{F}1=68.7$% on this task in [36]. However this required an elaborate use of an ensemble of task specific ResNet-18 models.

**Fig. 6.**
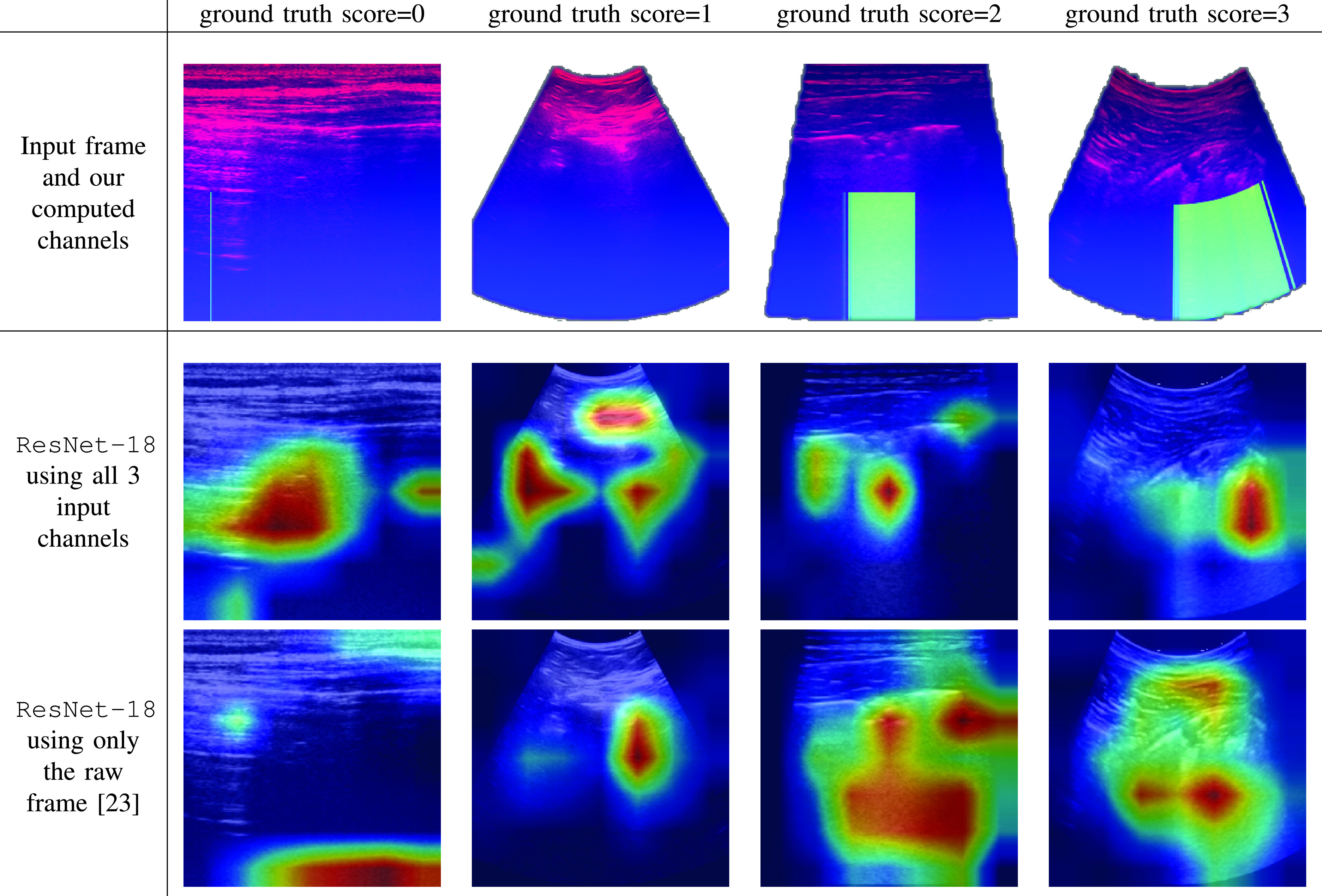
**Visualizing predictions using GradCAM.** Visualizing regions in the frame that most influence the model’s prediction. Top row: Overlay of the raw input frame and our estimated pleural line and vertical artifacts masks. Middle row: visualization of correct classifications by our framework– when all input masks are used. Bottom row: visualization of misclassifications by [23]– the same DNN architecture when only the raw input frame is used without the additional input masks.

We further used the GradCAM method [37] to visually inspect the predictions of ResNet-18 models. Fig. 6 shows a visual comparison of correct classifications by our framework to misclassifications of the baseline of [23]. That is, using the same deep neural architecture (ResNet-18), our model uses all three input channels whereas the baseline [23] uses only the raw frame. The first column shows a frame captured by a linear probe of a healthy patient (score $=0$). Our model (second row) attends well to the clear region below the pleural line and to the A-lines shown on the left part of the frame. Note that despite the thin vertical artifact falsely detected in the frame, the trained model was able to compensate and ignore it. In contrast, the baseline model falsely predicts severity score $=2$ and attends to irrelevant regions as the exterior tissue at the top of the frame or the void at the bottom. The second column shows a frame with score $=1$, misclassified by the baseline as score $=0$. Our model attends to the pleural line region, which holds important information for the score $=1$ class. In the third column is a frame labeled as score $=2$. The baseline model misclassified it as score $=3$. The GradCAM visualization shows how our model focuses on the vertical artifacts– thanks to the focused input mask, while the baseline model “spreads” all over the bottom part of the frame. Visualizing results for the most severe case, with score $=3$, on the fourth column, we see that our model successfully focuses on the wide “white lung” region below the pleural line. In contrast, the baseline model attends to irrelevant regions above the pleural line and thus misclassify this frame as score $=2$.

These visualizations suggests that our framework, namely, providing a model for LUS analysis with additional pleural line and vertical artifacts masks, is able to effectively steer the model to the relevant regions of the frame: It is able to inspect the pleural line and suspicious regions inside the lung cavity, while paying less attention to the tissue above the pleural line.

### C. Ablation Study

#### 1) Input Channels:

To show the complementary nature of the two additional input channels, we performed an ablation study. We used the same deep neural architecture, namely ResNet-18, and trained it using different combinations of input channels: Once with only the vertical artifacts masks and once with only the pleural line mask. Rows 3–6 of Table I show the performance of these trained ResNet-18 models. Adding only one additional channel (either vertical artifacts or pleural line channel) helps to increase performance by $\sim 2\%$ showing that these channels do contain useful information and the model is able to take advantage of it. However, when combining both channels ( $3^{rd}$ row in Table I) performance increase dramatically to $\text{F}1=68.8$%, significantly exceeding previous methods. Nevertheless, these additional channels cannot replace the raw input frames completely, as suggested by the $6^{th}$ row of the table. Discarding the raw input frame significantly degrades performance to merely $\text{F}1=45.1$%.

In their work on LUS classification [38] also considered incorporating domain knowledge using relevant semantic segmentation maps as inputs. However, their VGG-Seg model performs worse than their baseline VGG despite the additional semantic information. It seems that overriding the raw input channel in favour of semantic information prevents the model from compensating for inevitable inaccuracies in the input masks. In contrast, we leave the raw LUS frame intact as one of the input channels and only *augment* it with additional domain specific knowledge.

#### 2) Choice of Backbone:

Our proposed framework for training DNN models for LUS data is not restricted to any specific DNN architecture, and in fact allows for flexible choice of DNN architecture based on the requirements of the downstream task. We further experimented with different image classification DNN architectures for the task of COVID-19 severity classification within our framework. We compared light-weighted MobileNetV2 [30] and MobilenetV3 [39], as well as the classic VGG16 [40] architectures. Rows 7, 11 and 15 of Table I show the performance of these models using our framework (all input channels). Despite the fact that these are “general-purpose” image classification architectures that were not tailored to the idiosyncrasies of LUS, they perform on-par and even better than the specifically designed CNN-Reg-STN architecture of [23]. We additionally verified that all these different DNN models perform well thanks to the use of our framework, namely, utilizing all 3 input channels. When removing either the vertical artifacts or the pleural line channels, performance of all DNN models degrades significantly, in the same manner observed for ResNet-18 DNN model in §IV-C.1.

We note that even the light-weighted MobileNetV2 and MobileNetV3 models perform very well. This is in contrast to the findings of [38] that reported a significant drop in performance when using a “mobile” architecture for LUS classification. When having no access to domain knowledge thin mobile architecture are indeed likely to fail to extract meaningful features from the raw LUS frames. In constrast, in our framework, domain knowledge is easily accessible via the additional input channels making it easy to exploit even for light-weighted models.

Finally, when using a very deep DNN architecture, e.g., ResNet-101 (rows 19-22), performance degrade significantly: the training set is too small for this very large model (42M parameters compared to 11M of ResNet-18 or merely 1.5M of MobileNetV3). Hence, despite the fact that our framework is independent of the choice of DNN, the downstream task and the size of the training set may affect the performance of the chosen DNN architecture and may dictate the preference of one architecture over another.

### D. Effect of Training Set Size

**Fig. 7.**
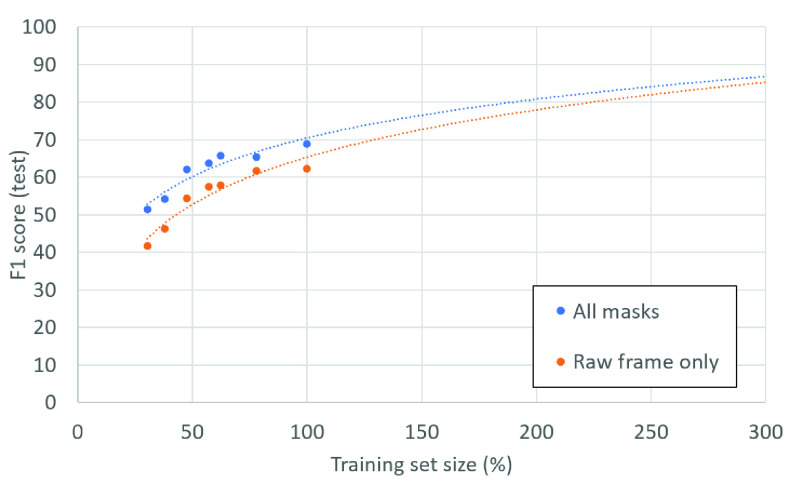
**Performance as a function of training set size:** F1 scores of ResNet-18 backbone trained with all masks (blue), vs. trained using the raw frames only (orange), as a function of training set size. Extrapolating the trend shows the gap between the two approaches diminishes as significantly more training data is introduced.

Although our masks do not add any external information that is not already “in the pixels”, explicitly extracting it for the network to use, makes the finetuning process efficient, quick and robust. We postulate that it would require substantially more labeled training examples for deep networks to automatically distill this specific domain knowledge directly from the “raw” pixels. To experimentally validate this assumption, we sub-sampled the training set, at a patient-level, and trained ResNet-18 backbone - once using our framework with all additional input masks, and once using only the raw input frames. The graph in Fig. 7 shows the F1 score (Settings 1) on the fixed test set as a function of the training set size, and an extrapolation of the trend. It can be seen that the gap between our framework and the baseline diminishes as the training set size increases. However, it seems like it requires a *significantly* larger training set (roughly ${\times }3$ larger) to bridge the gap between the baseline and our proposed framework. Obtaining these amounts of labeled data, especially in the medical imaging domain, is extremely challenging, highlighting the practical benefit of our framework.

Moreover, it can be seen from Fig. 7 that ResNet-18 trained with our framework was able to achieve $\text{F}1\sim 62.2\%$ by training on only ${\sim }50\%$ of the data compared to the same network naïvely trained on all the raw frames of the training data.

This result highlights the ability of our framework to overcome the challenge of limited training data in the domain of LUS; it reduced the amount of training data needed to train the network adequately.

### E. Vertical Artifacts Masks

Our approach for vertical artifacts detection is extremely simple yet, our experiments suggest it is still effectively guiding the downstream model in the right direction.

To get a sense of how informative our masks are we measured the relative width our detected vertical artifacts span, per frame, in the ICLUS dataset. To avoid noisy frame labeling we used the restricted test set of *Settings 2* with $K=7$. Recall that [6] defined the scores such that when vertical artifacts are detected it usually suggests that the frame should not be scored 0, and the wider these artifacts the more severe the condition is. Table II shows the relative width of vertical artifacts detected by our method per-frame. We can see that there is a distinction between frames with score $=0$,1 and those with score $=2$,3. However, the variance is quite large, highlighting the limitations of our simplistic approach.

**TABLE II:** Distribution of Detected Vertical Artifacts According to COVID-19 Severity Score of [6]: The Table Shows the Relative Frame Width per Frame (Std) the Detected Vertical Artifacts Span, and Their Average Number. Score $=0$ Is Healthy While Score $=3$ Indicates Acute Respiratory Condition. Vertical Artifacts Usually Indicate a Pathological Condition of the Lung, and Thus Appear More as the Score Increases

score	0	1	2	3
Width [%]	0.69±6.0	0.05±0.48	3.27±6.89	13.86±12.14
# B-lines	0.11± 1.41	0.10± 1.65	1.57± 4.40	7.36± 6.94

Since B-lines visualization is strongly dependent on the imaging settings and utilized hardware, traditional severity assessment by *counting* B-lines tends to be very subjective, compared to relative span suggested by [6]. Nevertheless, Tab. II also reports counts of vertical artifacts that leads to similar conclusions.

Our simple approach aims at highlighting any vertical artifacts, not just B-lines, consequently, using the Radon-based method of [24], [25] for B-lines detection, attained only $\text{F}1=67.6$% [41].

## V. Semantic Segmentation of COVID-19 Markers

To further demonstrate the applicability of our framework, we tested it on a different type of task: training a semantic segmentation DNN to segment LUS frames.

### A. Data

**Fig. 8.**
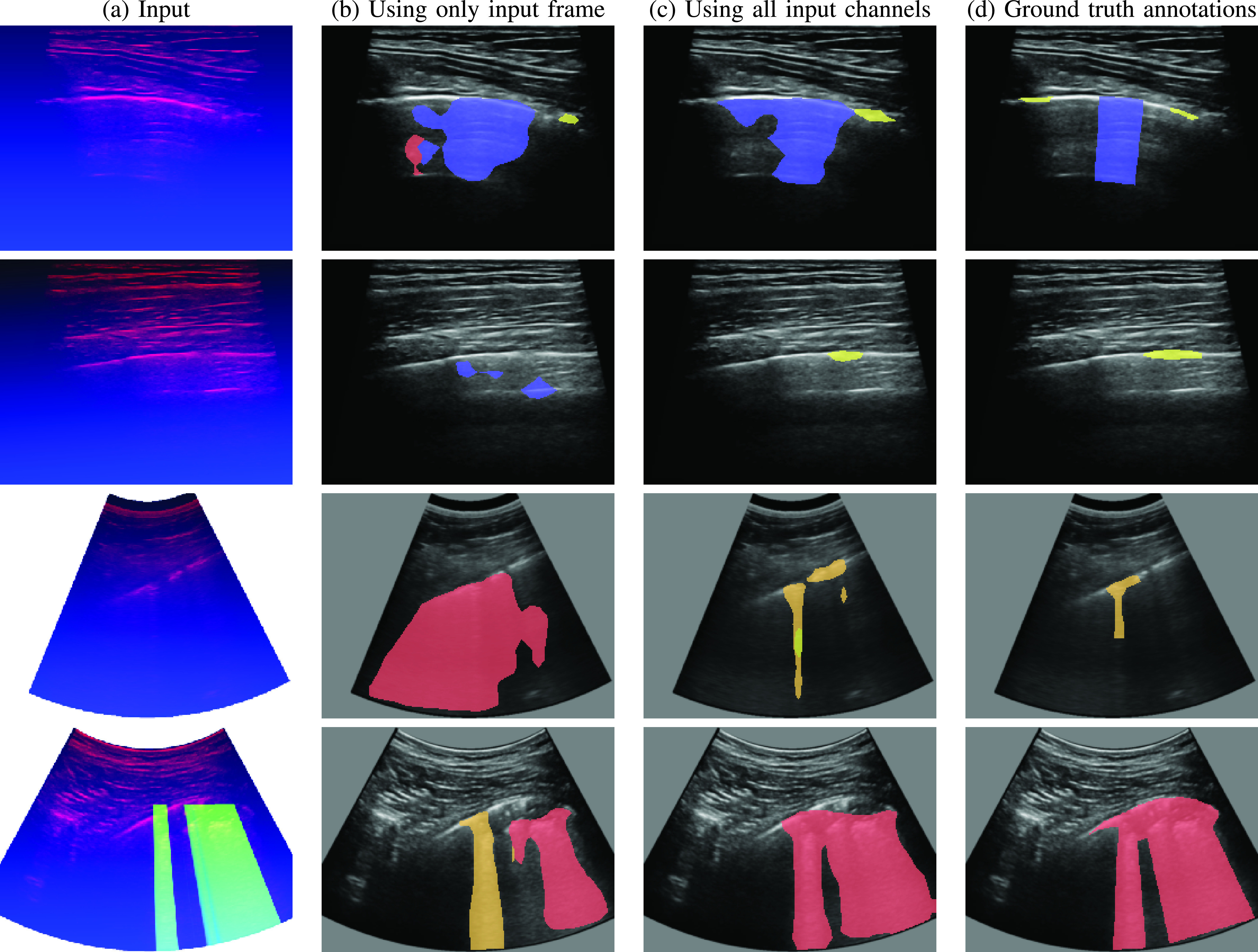
**Semantic segmentation:** Pixels indicating score $=0$ are annotated blue, score $=1$ in yellow, score $=2$ in orange and score $=3$ are annotated red. Note that the gray pixels outside the LUS scan are ignored. (a) Input frame and the additional channels computed by our framework. (b) Semantic segmentation results of DeepLabV3++ model utilizing only the raw input frames (as in [23]), Cat. Dice $=0.64$. (c) Segmentation results of the same DeepLabV3++ architecture utilizing all three input channels, Cat. Dice $=0.70$. (d) Ground truth annotations.

In addition to per-frame COVID-19 severity score annotations, the ICLUS dataset [23] provides detailed pixel-level annotations for the biomarkers indicative of each score. These detailed semantic annotations were provided for 2,154 frames across 33 patients, of which 1,602 frames were captured using convex probes and 552 using linear probes (note that we are using an updated view of the ICLUS dataset with more annotations compared to [23]). We further split the data into train and test sets according to the same patient-level split used in §IV with 1,601 (1,237 convex, 364 linear) training frames. For the frames in the training set, relative pixel-level occurrences for score 0, 1, 2 and 3 are 3.8%, 0.1%, 2.0% and 3.2% respectively. For the test set the relative occurrences are 5.6%, 0.2%, 1.6% and 4.7% respectively. Notably almost 90% of the pixels do not display clear characteristics of any specific class (severity score) and are, therefore, labeled as background. Unlike [23] we treat pixels outside the LUS field of view as “ignore” and discard them completely from the training and evaluation (e.g., gray pixels in Fig. 8d).

### B. Results

We used our framework to finetune a DeepLabV3++ semantic segmentation model with ResNet-50 backbone that was pre-trained on a subset of MS-COCO dataset [33] to predict a per-pixel severity score according to the semantic annotation of the ICLUS dataset. Table III shows the segmentation performance of our trained model measured, similar to [23], by accuracy across all categories (Acc.), the Dice coefficient for the union of COVID-19 related scored vs. background (Dice), and the mean Dice across scores 0, 2 and 3 (Cat. Dice). Score $=1$ is omitted due to its under representation in the pixel annotations. It is worthwhile noting that due to the disproportionate size of the background class ( $\sim 90$%) the accuracy and the Dice measures are quite biased and do not reflect well the performance of the model on the relevant COVID-19 classes. In contrast, the category Dice (Cat. Dice) measure focuses on the relevant labels and reflects better the performance of the model on this specific task.

**TABLE III:** Semantic Segmentation Results: Segmentation Performance Measured by Accuracy Across All Categories (Acc.), the Dice Coefficient for the Union of COVID-19 Related Scored (Dice), and the Mean Dice Across Scores 0, 2 and 3 (Cat. Dice) as in [23]. Accuracy and Dice Scores Are Heavily Biased Towards the Dominant Background Class, While Cat. Dice Reflects Better the Performance on the Relevant Annotated Pixels

Model		Input			Acc.	Dice	Cat. Dice
DeepLabV $3++\ast $ (Royetal.)		✓			0.95	0.71	0.62
Ensemble $\ast $ (Roy etal.)		✓			0.96	0.75	0.65
DeepLabV $3++$ (ours)		✓	✓	✓	0.93	0.76	0.70
Ablation	DeepLabV $3++$	✓			0.92	0.72	0.64
	DeepLabV $3++$	✓		✓	0.93	0.74	0.70
	DeepLabV $3++$	✓	✓		0.93	0.74	0.68
* Note that Roy *et al*. [23] trained and evaluated only on convex frames, while our method used both linear and convex.							

We train the same DeepLabV3++ architecture, once using our framework with all input channels (e.g., the raw frame, vertical artifacts and pleural-line channels) and once using only the raw input frame as in [23]. We trained and evaluated on frames obtained from both convex and linear probes. Adding the linear frames slightly improves the Cat. Dice from 0.62 reported by [23] to 0.64 ( $1^{st}$ and $4^{th}$ rows of Tab. III). However, adding the additional input channels boost the Cat. Dice score even further to 0.70 ( $3^{rd}$ row of Tab. III).

Fig. 8 shows four examples of input frames, the corresponding predicted segmentation masks and the ground truth annotations. The color of the segments indicate their corresponding COVID-19 severity score from blue for score $=0$ through yellow, and orange all the way to red corresponding to scores 1, 2 and 3 respectively. Note how our trained model is capable of handling both linear (first two rows) as well as convex (bottom rows) frames, as opposed to [23] that was restricted to convex frames only. Providing a model with the domain specific knowledge in the form of the location of the pleural line allows it to better detect small discontinuities corresponding to the challenging score $=1$ category (first two rows). Explicitly providing the model with information on vertical artifacts allows it to better classify “white lung” regions as score $=3$ rather than score $=2$ ( $4^{th}$ row).

### C. Ablation Study

To show the impact of the two additional input channels, we performed an ablation study. We used the same deep neural architecture, namely DeepLabV3++, and trained it using different combinations of input channels: Once with only the vertical artifacts masks and once with only the pleural line mask. The bottom two rows of Table III show the performance of these two trained DeepLabV3++ models. Adding only the pleural line channel already increase the Cat. Dice score to 0.70 leaving only a difference of 0.02 on the Dice score compared to the model trained using all three channels, showing the power of pleural-line location information for the localization of relevant biomarkers. In contrast, adding only the vertical artifacts channel resulted with a less prominent performance gain: Cat. Dice increased to only 0.68. This is probably due to the inaccuracies in B-line detection of our method already discussed in §IV-E.

## VI. Conclusion

In this work we introduced a framework for combining the power of deep neural networks with prior domain knowledge specific to LUS resulting in a fast and efficient way of training DNNs on LUS data. The key insight is to explicitly provide domain-specific knowledge to the models. However, instead of incorporating this knowledge in the form of elaborate and task-specific DNN architecture design, we propose to introduce it as part of the input data. In the context of LUS we demonstrated that informing the model of the location of the pleural line and the presence of vertical artifacts, such as B-lines and “white lung”, can significantly improve performance. Moreover, it allows to treat frames captured by either linear or convex probes in a unified manner– using a *single* DNN for both types of frames. We exemplified the applicability of our framework on COVID-19 severity assessment, both on the task of LUS frame classification as well as the task of LUS semantic segmentation.

Although our masks do not add any external information that is not already “in the pixels”, explicitly extracting it for the network to use, makes the finetuning process efficient, quick and robust, and can be done on much smaller datasets. Experiments suggest that it would require significantly longer training time and substantially more labeled training examples for deep networks to *automatically* distill this specific domain knowledge directly from the “raw” pixels.

Our proposed framework is, therefore, more widely applicable to LUS than COVID-19 severity prediction. The framework can be used not only to train different DL architectures, but to address other challenges in the analysis of LUS frames. Moreover, our framework does not rely on any *specific* implementation of pleural line or vertical artifacts detection algorithms and allows for incorporating other domain knowledge e.g., A-lines, and more, in a similar manner.
